# Topical Treatments for Rare Genetic Dermatological Diseases: A Narrative Review

**DOI:** 10.3390/ph18111762

**Published:** 2025-11-19

**Authors:** Beatriz de Araujo Oliveira, Ana Torres, Eduardo Ricci-Júnior, Isabel F. Almeida, Mariana Sato S. B. Monteiro

**Affiliations:** 1 Laboratório de Desenvolvimento Galênico, Faculty of Pharmacy, Federal University of Rio de Janeiro (UFRJ), Rio de Janeiro 21941-971, Brazil; beatriiz.araujo98@gmail.com (B.d.A.O.); eduardoriccijunior@gmail.com (E.R.-J.); mari-sato@hotmail.com (M.S.S.B.M.); 2 UCIBIO—Applied Molecular Biosciences Unit, Department of Drug Sciences, Faculty of Pharmacy, University of Porto, 4050-313 Porto, Portugal; up201706122@up.pt; 3 Associate Laboratory i4HB, Institute for Health and Bioeconomy, Faculty of Pharmacy, University of Porto, 4050-313 Porto, Portugal

**Keywords:** rare genetic diseases, topical treatments, orphan diseases, dermatological diseases, compounding medicines

## Abstract

Rare diseases are conditions that affect up to 65 people per 100,000 individuals. They are also known as “orphan diseases”, because they attract limited interest from researchers and pharmaceutical industries. Epidermolysis bullosa (EB), ichthyosis, Hailey–Hailey disease (HHD), Darier disease (DD), erythrokeratoderma, porokeratosis, inflammatory linear verrucous epidermal nevus (ILVEN) and piebaldism are examples of rare genetic skin diseases with few approved treatments. Topical treatments are the principal approach for rare dermatological diseases, and it can be useful to manage the symptoms or the patophysiology of these diseases. This study aimed to conduct a comprehensive review of the topical treatments of EB, ichthyosis, HHD, DD, erythrokeratodermias, porokeratosis, ILVEN, and piebaldism. The search was performed across the SciELO, MEDLINE^®^/PubMed^®^, Embase and Cochrane databases. This review identified porokeratosis, EB, and congenital ichthyosis as the rare genodermatoses with the highest number of reported studies and topical treatment options. In contrast, conditions such as piebaldism, erythrokeratodermia, and HHD have fewer reported topical interventions. For most rare genetic dermatological diseases, treatment aims to improve quality of life and control clinical signals and symptoms. Creams, gels, and ointments are frequently used as the main pharmaceutical approaches, and several pharmacological classes are employed, including keratolytics, retinoids, vitamin D analogs, topical corticosteroids, calcineurin inhibitors, and cytotoxic or antiproliferative agents. This review highlights the potential of off-label use of topical therapies as cost-effective alternatives in the treatment of rare genetic skin disorders. It also reinforces the critical role of compounded medicines in allowing for dose optimization, drug repurposing, and formulation adjustments, personalizing treatment to achieve improved therapeutic outcomes.

## 1. Introduction

Rare diseases are chronic conditions generally affecting no more than 65 individuals per 100,000 people. It is estimated that there are between 6000 and 8000 different types of rare diseases worldwide, and approximately 300 million people are affected by these globally [1,2]. Patients with these conditions typically have complex needs in terms of treatment and monitoring, which consequently requires continuous care, as well as an integrated and multidisciplinary approach [3,4]. Due to their low prevalence and the limited clinical and therapeutic data available, these diseases are frequently underdiagnosed and neglected by healthcare systems.

Rare dermatological disorders include more than 800 diseases, affecting over 6.8 million patients worldwide [5]. Among them, those of genetic origin are particularly significant, as they often manifest early in life, follow a chronic and progressive course, and require complex management [6]. The most well-known rare genetic dermatological conditions can be classified into distinct groups based on their pathophysiology. These include genetic disorders affecting epidermal cohesion or adhesion, such as epidermolysis bullosa (EB) [7], Hailey–Hailey disease (HHD) [8] and Darier disease (DD) [9]; genetic disorders of keratinization, including ichthyosis [10], erythrokeratodermias [11] and porokeratosis [12]; Nevi and cutaneous mosaic disorders, such as inflammatory linear verrucous epidermal nevus (ILVEN) [13]; and genetic pigmentation disorders, such as piebaldism [14].

### 1.1. Epidermolysis Bullosa

**Figure 1:**
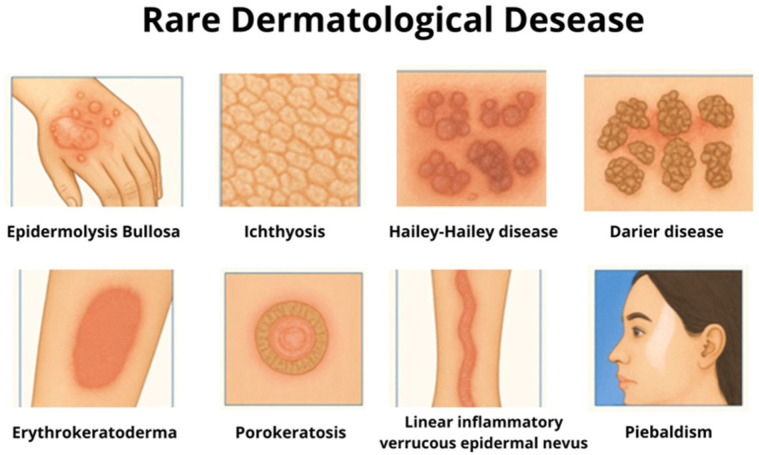
Representative clinical manifestations of rare dermatological diseases are included in this review. From left to right, top row: Epidermolysis bullosa (EB), characterized by skin fragility and blister development; Ichthyosis, characterized by hyperkeratosis; Hailey–Hailey disease, characterized by erosive lesions and recurrent vesicles; and Darier disease (DD), characterized by papules and plaques. Bottom row: Erythrokeratoderma, characterized by erythematous and hyperkeratotic plaques; Porokeratosis, characterized by annular plaques; Inflammatory linear verrucous epidermal nevus (ILVEN), characterized by erythematous and verrucous papules and Piebaldism, characterized by congenital depigmented macules. Source: Created by the authors using BioRender^®^ and Canva (2025). Created in BioRender. MONTEIRO, M. (2025) https://BioRender.com/le4fv2s (accessed on 16 November 2025).

EB is a group of phenotypically distinct genodermatoses, with a prevalence of 11.1 per one million live births in the United States [15]. EB can be either hereditary or acquired, and it is characterized by skin fragility and blister formation in response to mechanical trauma, which can also affect the mucous membranes (Figure 1). Since severe forms of the disease are typically associated with a high risk of infection, the neonatal mortality rate is high [7,16].

EB can be classified into four main types based on the histopathological profile of the blisters: simplex, junctional, dystrophic, and kindler syndrome. In simplex EB, blister formation is superficial and non-scarring. The occurrence of blisters tends to decrease with age [16] and result from mutations in keratin K14 or K5 [17]. In junctional EB, the blisters are deeper and affect most of the body surface. Death may occur before the first year of life. However, once complications are managed, the condition tends to improve with age [18]. In dystrophic EB, the blisters are also formed deeply between the dermis and epidermis, leading to scarring and often the loss of limb function [18]. Pruriginous EB is a subtype of dystrophic EB, characterized by itchy or lichenified lesions associated with scarring, typically confined to the shins and forearms [19]. Recessive dystrophic EB is the most severe form and is attributed to mutations in the *COL7A1* gene, which encodes type VII collagen, a structural protein of the skin [20]. Because skin integrity is highly compromised in this EB subtype, characteristic clinical manifestations such as severe and chronic blistering, mitten deformities of the hands and feet, mucosal and internal organ involvement, and an increased risk of developing aggressive squamous cell carcinoma arise [21,22].

Kindler syndrome is considered a mixed form of the other types, where blistering occurs at the dermo-epidermal junction. It is often associated with photosensitivity, skin atrophy, intestinal inflammation, and mucosal stenosis [19]. Diagnosis, generally, occurs in the neonatal period, although lesions may not appear in some individuals until adolescence or later, delaying accurate diagnosis until adulthood [22].

### 1.2. Hailey–Hailey Disease

HHD is a rare autosomal dominant disorder, with a prevalence of 1:50,000 and no gender or race predilection [23]. It causes the appearance of recurrent vesiculobullous and erosive lesions, mainly in body areas subjected to friction, such as the armpits, neck, and groin (Figure 1) [24]. The disease results from a mutation in the *ATP2C1* gene, which impairs the function of a protein responsible for balancing calcium gradient in skin cells. The cytosolic accumulation of Ca^2+^ decreases mitochondrial ATP synthesis, disrupts actin cytoskeleton organization, and increases oxidative stress. These events impair keratinocyte proliferation, differentiation, and compromises intercellular adhesion in the epidermis, prompting lesion formation [23]. Clinically, lesions are often exacerbated by heat, sweating, friction or secondary infection, and can impact quality of life [23,24].

### 1.3. Darier Disease

DD is a rare autosomal dominant genodermatosis, with a prevalence ranging from 1:100,000 to 1:30,000 in northern European populations, affecting men and women equally [25]. DD is caused by mutations in the *ATP2A2* gene, which produces the calcium pump protein SERCA2 (sarco/endoplasmic reticulum Ca^2+^-ATPase 2). This protein is essential for maintaining intracellular calcium homeostasis and plays a critical role in keratinocyte adhesion and differentiation. Dysfunction of SERCA2 compromises cell cohesion in the epidermis. Clinically, DD presents as papules that coalesce into plaques, especially in seborrheic areas such as the scalp, forehead, chest, and back (Figure 1). Symptoms are chronic and recurrent, tend to worsen with heat, sweating, friction and secondary infections. In addition to cutaneous manifestations, patients may experience neuropsychiatric symptoms such as depression, anxiety, and cognitive disturbances [9].

### 1.4. Congenital Ichthyosis

Ichthyosis refers to a group of hereditary and acquired disorders of keratinization and can be further subclassified as non-syndromic—if it only affects the skin—or syndromic—if it simultaneously affects other organs [26]. Non-syndromic ichthyoses include common ichthyoses, such as ichthyosis vulgaris (IV) and recessive X-linked ichthyosis (RXLI), autosomal recessive congenital ichthyosis (ARCI), and keratinopathic ichthyosis [27,28].

IV, the most common hereditary form of ichthyosis [29], is linked to filaggrin (FLG) mutations, that lead to the stratum corneum (SC) disruption, thereby increasing skin permeability, transepidermal water loss (TEWL), and susceptibility to allergens and microorganisms [30]. It has a milder phenotypical presentation than other types and is characterized by fish-like brownish dry, scaly and thickened skin [31]. RXLI is the second most common type and mainly affects males [32]. XLRI manifests in the immediate neonatal period as generalized, large, slightly adherent, and lightly pigmented scaling, affecting the scalp, preauricular area and posterior neck. The most common associated extracutaneous findings include corneal opacities, epilepsy, and electroencephalographic abnormalities [33]. RXLI is caused by deficient activity of the steroid sulfatase (STS) enzyme, which is attributed to complete or partial deletions of the *STS* gene [34]. Even though it is less common, there are also syndromic types of ichthyoses, which are characterized by mutations in genes involved in the cholesterol biosynthesis pathway. Syndromes such as Ichthyosis folliculareis–alopecia–photophobia (IFAP), X-linked dominant chondrodysplasia punctata (CDPX2) and Congenital hemidysplasia with ichthyosiform erythroderma and limb defects (CHILD Syndrome) result from mutations in the MBTPS2, EBP and NSDHL genes, respectively. These mutations compromise correct cholesterol synthesis and lead to the accumulation of toxic sterols. Alterations in cholesterol metabolism result in defects in the SC lipid envelope, leading to increased permeability to water and ions, raised pH, activation of proteases that degrade corneodesmosomes, and processing enzymes that assemble the SC lipidic envelope. Increased SC permeability also activates pro-inflammatory cytokines, which induce abnormalities in keratinocyte proliferation and differentiation. Furthermore, sterol precursors accelerate the degradation of 3-hydroxy-3-methylglutaryl coenzyme A (HMG CoA) reductase, the enzyme limiting cholesterol synthesis [35].

ARCI typically exhibit the most severe phenotypic presentation among non-syndromic common ichthyoses. It is characterized by redness, blistering, and skin thickening, often associated with infections due to the decreased skin barrier (Figure 1) [35,36]. ARCI include several subtypes, such as lamellar ichthyosis (LI), non-bullous congenital ichthyosiform erythroderma, X-linked ichthyosis, and harlequin ichthyosis (HI), which presents the most severe phenotype [37,38]. LI is characterized by hyperkeratosis and desquamation, leading to thick keratin scales across the body, xerosis, and skin rigidity. This rigidity can prevent proper eyelid closure, resulting in ectropion and ocular complications [39,40]. On the other hand, HI is characterized by a severe collodion membrane that develops, within a few weeks, generalized scaling with severe erythroderma [26]. ARCI is caused by mutations in one of 12 genes involved in epidermal differentiation. Approximately 30% of cases are attributed to loss-of-function mutations in the *transglutaminase 1* (*TGM1*) gene, which produces a key enzyme involved in the organization of the cornified envelope [10,41]. However, a gap remains in the understanding of the pathophysiological pathways of ARCI, which represents a challenge for medical treatment and the development of pharmacological solutions [37]. Patients’ quality of life may also be affected by the social and psychological consequences of the disease [38].

### 1.5. Erythrokeratodermias

Erythrokeratodermias are a group of rare genodermatoses characterized by the presence of hyperkeratotic plaques associated with areas of erythema (Figure 1). These conditions exhibit both clinical and genetic heterogeneity and are classified into two main subtypes: Progressive Symmetrical Erythrokeratoderma (PSEK) and Erythrokeratoderma Variabilis (EKV) [42,43].

PSEK begins in childhood and presents as non-migratory, symmetrical, erythematous, hyperkeratotic plaques. These lesions range in color from reddish orange to brown and show marked peripheral erythema. A typical sign is the symmetry of the lesions, which affect the knees, elbows, hands, and feet [43]. Genetically, PSEK shows considerable phenotypic variability and is predominantly congenital in an autosomal dominant manner. Although its pathophysiology is not fully understood, mutations in the *LOR* gene, located on chromosome 1q21, have been identified in some cases. The *LOR* gene involves loricrin, a structural protein of the cornified cell envelope, a highly resistant protective layer formed in epidermal cells during keratinization. Loricrin plays a fundamental role in the skin barrier, being one of the main proteins that contribute to skin rigidity and impermeability. It is also involved in the formation of keratohyalin granules, structures found in the granular layer of the epidermis that assist in organizing and compacting proteins during the cornification process [43,44].

EKV is distinguished by the combination of two types of skin lesions: fixed, well-demarcated, symmetrical hyperkeratotic plaques that persist in specific areas, and transient, migratory erythema that shifts location over the course of hours or days. These manifestations may occur simultaneously or alternate throughout an individual’s lifetime. The disease typically begins in childhood, although late-onset cases have been reported. Facial involvement, especially on the cheeks is common, as is the involvement of flexural areas such as the axillae, groin, neck, and trunk [42]. EKV exhibits marked phenotypic variability, even among members of the same family. Genetically, EKV is associated with mutations in the *GJB3* and *GJB4* genes, which produce connexin proteins. These proteins are crucial components of gap junctions, mediating communication between keratinocytes. Their functions are essential for intercellular communication and epidermal homeostasis [45].

### 1.6. Porokeratosis

Porokeratosis (PK) is a rare genodermatosis characterized by annular or irregular skin lesions with raised, well-demarcated borders and a central area of epidermal atrophy (Figure 1). These lesions most commonly develop in sun-exposed areas, such as the arms and legs, but can appear anywhere on the body [46]. PK is primarily caused by inherited pathogenic variants in specific genes. However, symptoms usually require an additional cause, often related to external factors such as ultraviolet (UV) radiation, immunosuppression, or skin trauma [12]. Several clinical subtypes of porokeratosis have been described, with Disseminated Superficial Actinic Porokeratosis (DSAP) and Mibelli-type porokeratosis being the most prevalent. Genetic studies have identified mutations in genes of the mevalonate pathway, specifically *MVK* (mevalonate kinase), *PMVK* (phosphomevalonate kinase), *MVD* (mevalonate diphosphate decarboxylase) and *FDPS* (farnesyl diphosphate synthase [47]. Consequently, cholesterol biosynthesis is compromised, and the expression of differentiation markers, keratin 1 and involucrin, is decreased, affecting skin barrier integrity [48]. The biosynthesis of dolichol and ubiquinone is also compromised, which impairs protein prenylation and post-translational modifications. Additionally, the phosphorylation of lipid intermediates is affected, leading to the activation of pyrin and the inflammasome. These disruptions result in reduced levels of isoprenoids, which are identified as the pathological mechanism inducing the proinflammatory phenotype of this disease [49,50].

The histopathological findings of porokeratosis are the cornoid lamella, a thin column-like structure of parakeratotic cells that represent abnormal keratinization. Management focuses on symptom control and the prevention of complications [12]. Due to a documented risk of malignant transformation, particularly into squamous cell carcinoma, long-term dermatologic observation is recommended [51].

### 1.7. Inflammatory Linear Verrucous Epidermal Nevus

ILVEN is a rare clinical variant of verrucous epidermal nevus that typically begins at birth or during childhood [13]. It is a rare condition characterized by recurrent inflammatory phenomena, resembling chronic eczematous or psoriasiform dermatitis. Clinically, it presents as erythematous and verrucous papules associated with intense pruritus (Figure 1). The lesions are persistent and primarily affect the lower limbs [52,53,54]. ILVEN is also believed to be associated with the upregulation of key inflammatory mediators, including interleukin (IL)-1, IL-6, tumor necrosis factor-alpha (TNF-α), and intercellular adhesion molecule 1. Infection may act as a trigger for inflammation in ILVEN [55]. Moreover, ILVEN can persist throughout life, and its chronic and progressive nature may lead to physical and psycho-emotional consequences, impacting in patients’ quality of life. Due to the potential risk of malignancy, long-term monitoring is necessary [13].

### 1.8. Piebaldism

Piebaldism is a rare autosomal dominant disorder that affects the migration and development of melanocytes. At birth, piebaldism is distinguished by a white frontal hair patch, also known as poliosis, and symmetrical depigmented macules on the skin [14]. The skin depigmentation is quite distinctive, characterized by strict symmetry, predominantly affecting the face, anterior chest and abdomen, arms, forearms, legs, and thighs. The lesions usually remain stationary or show mild and limited progression, with proportional enlargement as the patient grows (Figure 1) [14]. Although generally benign and limited to skin, piebaldism can be socially disabling for affected individuals [56].

Piebaldism is, in most cases, characterized by a mutation in the *KIT* proto-oncogene [56], that leads to an abnormal distribution and reduced proliferation of melanoblasts during embryonic development. Consequently, the decrease in melanocytes differentiation results in melanin absence in the epidermis [57]. Furthermore, the abnormal migration of melanocytes from the neural crest causes leukoderma that affects the central forehead, frontal scalp, middle portions of the limbs, and central anterior trunk, where melanocytes are absent. Leukotrichia may also extend to axillary and pubic hair [57,58].

### 1.9. Topical Treatment of Rare Genetic Dermatological Diseases

The treatment of rare diseases faces major challenges, not only due to the difficulty and possible delay in diagnosis, but also because of the progressive, degenerative, and disabling nature of these conditions, along with the wide range of signs and symptoms they may present. Furthermore, treatment requires significant investment in research and the development of specific medications. Also, these drugs are used by a very limited number of individuals, consequently their production is expensive and scarce. Although such treatments are difficult to access, they are crucial for individuals with rare diseases, as they help to manage symptoms, prevent incapacitating clinical outcomes, delay disease progression or enable remission [59]. These patients often require personalized therapies adjusted to their specific needs, involving different pharmaceutical forms, dose adjustments, and drug combinations [60]. Therefore, the main goal of this study was to map the topical treatment options for these rare genetic cutaneous diseases gathering clinical evidence regarding the dose, posology, pharmaceutical form and efficacy outcomes, to support clinical decision-making. Additionally, this review aims to critically analyze the limitations of the studies and unravel the gaps in the literature to optimize future research.

## 2. Results

### 2.1. Search Strategy

The search using the keywords mentioned above yielded a total of 1070 articles across the databases from January 2013 to June 2025: 106 from MEDLINE/PubMed, 4 from SciELO, 881 from Embase and 88 from Cochrane. After removing 308 duplicate articles, the total number was reduced to 762 articles. Table 1 summarizes the number of articles found according to the consulted databases and the keywords used.

**Figure 2:**
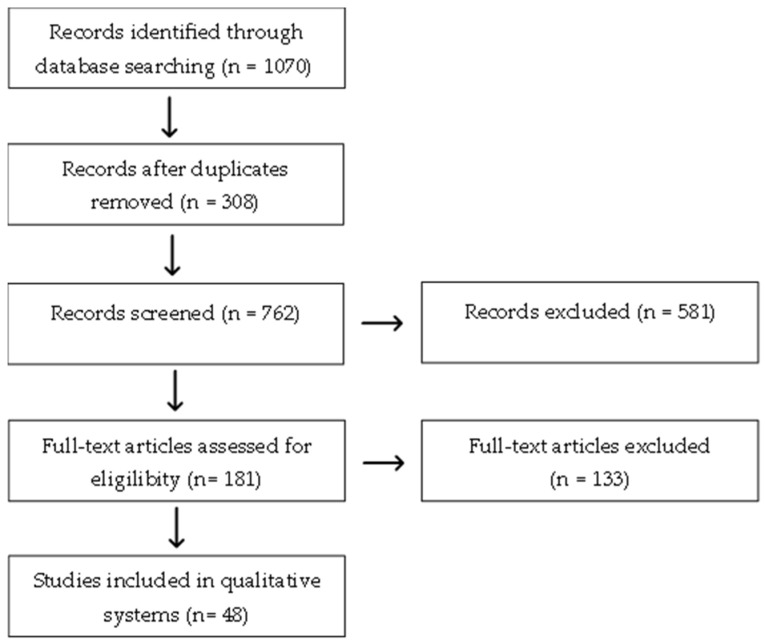
Flowchart summarizes the results of the literature search and selection process for studies on the topical treatment of rare genetic dermatological diseases. A total of 1070 records were identified through database searches. After removing 308 duplicates, 762 articles were evaluated and 581 were excluded for not achieved the inclusion criteria. The remaining 181 articles were assessed in full text and 48 were included in this review.

The initial screening found that, of the 762 remaining articles, 581 had to be excluded because they were part of the exclusion criteria. These criteria included: studies not related to the subject matter; opinion articles, editorials, letters to the editor, and conference abstracts lacking complete data; Preclinical studies (in vitro and/or in animal models), articles published in other languages other than Portuguese or English; Studies that employed both topical and systemic treatment, and articles outside the scope. This process left 48 articles eligible for this review (Figure 2).

Among the eligible articles, the distribution by disease was as follow: 18.8% (9 articles) addressed the topical treatment of EB, 4.2% (2 articles) focused on HHD, 10.4% (5 articles) discussed topical treatment of DD, 20.8% (10 articles) discussed topical treatment for Congenital Ichthyosis, 4.2% (2 articles) discussed topical treatment for Erythrokeratoderma, 27% (13 articles) focused on topical treatment for Porokeratosis, 12.5% (6 articles) covered topical treatment of ILVEN and 2.1% (1 article) focused on topical treatment of piebaldism (Figure 1).

### 2.2. Topical Treatment of Epidermolysis Bullosa (EB)

The treatment of EB focuses on care management, wound dressing techniques, and symptoms and pain management, as well as addressing psychological and social impacts. Topical therapies are central to EB management, aiming not only to alleviate symptoms but also to address underlying causes, control contributing factors, and prevent secondary infections using antimicrobial agents. Effective wound care involves creating an optimal healing environment adapted to the location and characteristics of each lesion, considering factors such as infection risk, exudate levels, and EB subtype [61]. Although the prognosis of this rare dermatological condition is relatively good, with blisters improvement with age, several topical therapies targeting EB have been explored over the years, ranging from basic wound care agents to advanced pharmacological approaches (Table 2). An important advancement in the management of EB was the approval of the topical gene therapy beremagene geperpavec (Vyjuvek^®^), indicated for patients with dystrophic EB caused by *COL7A1* mutations [62]. Furthermore, the therapeutic landscape is expanding with emerging gene therapies in clinical trials, such as the cell-based gene therapy approach (e.g., dabocemagene autoficel) and topical gene editing strategies, signaling a shift towards precision therapies development for EB [62,63].

Among these novel topical therapies, the recent approval of botanical formulations like birch triterpene gel (Filsuvez^®^) marks a significant milestone in EB management [63]. In a phase II study by Schwieger-Briel et al. (2017) [64], Oleogel-S10 (10% birch bark triterpenes) was evaluated for wound healing in patients with dystrophic EB. The results showed ≥95% epithelialization in 5 out of 12 wounds and a reduction in the mean wound closure time from 14 to 10.5 days, indicating its potential to accelerate healing [64]. In addition, a phase III, double-blind, randomized, vehicle-controlled trial by Kern et al. (2023) demonstrated a significantly higher rate of complete wound closure within 45 days in the Oleogel-S10 group (41.3%) compared to the control (28.9%), with a comparable safety profile [65]. The long-term efficacy and safety of Oleogel-S10 were further demonstrated by Murrell et al. (2025), showing sustained reductions in wound surface area, with a mean of 4.3% after 3 months, 5.9% after 12 months, and 3.7% after 24 months [66]. This may be due to the positive modulating of several mediators involved in the inflammatory phase of wound healing, namely IL-6, an important player in wound healing and epidermal barrier repair [67]. In fact, dry birch bark extract and its main triterpene component, betulin, have demonstrated anti-inflammatory and wound-healing properties. A transient increase in pro-inflammatory cytokines, namely IL-6 and IL-8, observed at the beginning of treatment is followed by a gradual attenuation during long-term administration. This suggests that an initial inflammatory stimulus may be a requisite for a subsequent anti-inflammatory and regenerative response. Moreover, both the birch bark extract and its components (betulin, lupeol, and erythrodiol) have been shown to enhance the migration of primary human keratinocytes, accelerate wound healing, and promote keratinocyte differentiation in vitro and in vivo processes, consequently restoring the skin barrier [64,65,66,67]. In vitro and in vivo studies suggest that betulin also has anticarcinogenic properties and induces apoptosis in different tumor cells [68]. Sunflower oil, which was used as a vehicle in this topical formulation, is rich in oleic, linoleic, and linolenic acids, which provide nourishing, emollient, and re-epithelializing properties, supporting its incorporation into creams at various concentrations [69,70].

Beyond the birch bark extract, further studies have explored the topical application of other substances that accelerate wound healing while delivering antimicrobial and anti-inflammatory effects. For instance, Paller et al. (2020) [71] conducted a phase III trial to evaluate the efficacy and safety of SD-101, a topical cream containing 6% allantoin in treating wounds and other skin lesions in patients with simplex, recessive dystrophic, or intermediate junctional EB. The SD-101 formulation includes allantoin along with multiple excipients: beeswax, butylated hydroxytoluene (BHT), cetyl alcohol, citric acid, cod liver oil, lanolin oil, methylparaben, propylene glycol, propylparaben, sodium lauryl sulfate, stearyl alcohol, ethylenediaminetetraacetic acid tetrasodium (EDTA), and purified water [71]. In preclinical studies, allantoin exhibited multiple wound healing effects, including anti-inflammatory and antimicrobial activity, as well as promoting tissue formation and differentiation, specifically by stimulating collagen deposition and epithelialization [71,72,73]. The study by Paller et al. (2020) suggested that SD-101 may promote faster wound closure compared to the vehicle, with tolerability [71]. The excipients have specific roles: Beeswax (5–9%) and cetyl alcohol (1%) enhance cream texture, BHT (0.01–0.03%) acts as an antioxidant, while citric acid adjusts the pH, methylparaben and propylparaben (each at 0.1%) function as preservatives, lanolin is an emollient, propylene glycol functions as a humectant in varying concentrations, sodium lauryl sulfate is a common surfactant, and stearyl alcohol (3%) is used in ointments, and EDTA (0.2%) acts as a chelating agent in various formulations [72].

According to data from the European Medicines Agency (2016), topical calcipotriol use was also reported for dystrophic EB, although details regarding the vehicle and concentration were not disclosed [74]. Subsequently, a phase II, randomized, double-blind, placebo-controlled, crossover clinical trial by Guttmann-Gruber et al. (2022) [75] in patients with dystrophic EB and *COL7A1* gene mutations demonstrated significant results. The topical application of calcipotriol 0.05 µg/g ointment led to a significant reduction in wound area (88.4% in the treated group compared to 65.5% in the placebo group). It also led to an improvement of pruritus, without significant adverse events [75]. Cathelicidin is a prominent antimicrobial peptide in human epithelial cells, capable of enhancing host defense, tissue repair, and wound closure. In response to skin infections, it is upregulated in the skin, exhibiting antimicrobial, antiviral, and antifungal activity [76]. The fact that vitamin D directly regulates cathelicidin has driven the investigation into the potential of topical calcipotriol in enhancing wound healing in dystrophic EB [77]. Calcipotriol can be compounded in concentrations of 0.005% or higher, provided the total amount does not exceed 100 g/week in ointments, gels, and lotions [76,77].

Moreover, clinical studies have explored topical agents with anti-inflammatory properties for milder EB presentations. For example, Wally et al. (2018) [78], in a randomized, placebo-controlled, phase II/III clinical trial, evaluated the effect of a 4-week treatment with 1% diacerein cream on blisters, reducing symptoms in 17 patients with EB simplex. A significant reduction in the absolute number of blisters was observed in the diacerein-treated group, with no adverse events being reported [78]. Building on these findings, Wally et al. (2013) [79] conducted a phase I, double-blind, randomized, placebo-controlled study using 1% diacerein cream in 5 patients with severe generalized EB. The treatment resulted in a significant reduction in the number of blisters within 2 weeks, and levels remained below baseline even after treatment discontinuation, with no adverse events reported [79]. Diacerein is an IL-1ß inhibitor. High levels of IL-1ß lead to the disintegration and collapse of the intermediate filament network, protein aggregation, and increased fragility to mechanical stress and osmotic shock. Therefore, therapeutic approaches that inhibit the IL-1β signaling pathway may be beneficial for patients with EB and EB simplex by reducing cellular inflammation and stabilizing the intermediate filament network after thermal shock [79,80]. Diacerein can be formulated in ointments, creams, or gels, at typical concentrations of 1% [80,81].

In addition to the wounds and associated inflammation, some EB patients may experience persistent itching and localized pain. Chelliah et al. (2018) showed that 3 EB patients treated with topical cannabidiol (CBD) oil presented fewer blisters and a shorter healing time for active blisters, with no adverse effects [82]. CBD is a naturally occurring compound found in industrial hemp and marijuana, both forms of cannabis. It has an affinity for the cannabinoid receptors CB1 and CB2 [83]. CB1 receptors, mainly found in brain and spinal cord nociceptors, are associated with cannabinoid-induced analgesia. CB2 receptors, expressed in lymphoid tissues, may modulate cytokine release and inflammation through CBD binding [84]. Nevertheless, CBD also acts as a direct agonist of vanilloid pain receptors, which are known for mediation pain perception, inflammation, and body temperature [85]. CBD alleviates EB symptoms by relieving chronic pain, modulating pruritus, and reducing inflammation [82,83,84]. Topical CBD formulations are available as oils, creams, and sprays, but they cannot be compounded [86].

While the previous explored agents were already approved for similar dermatological purposes, repurposing drugs commonly used for other routes of administration and different therapeutic applications have also been explored for EB. In this regard, Yasar et al. (2018) [87] reported, a case of a baby with EB whose lesions were treated with sterile gauze dressings and cream based in sterile petroleum jelly with 10% topical sucralfate, a drug originally approved the treatment of gastrointestinal ulcers. The treatment was applied once a day under occlusion for two weeks, resulting in nearly complete epithelialization [87]. Reports of the effects of topical sucralfate on wound epithelialization, along with its bacteriostatic properties, led to the evaluation of its efficacy as a topical agent for treating burn-like wounds in EB [87]. Sucralfate can be compounded as a powder, cream, ointment, suspension, or gel, with typical concentrations ranging from 1% to 10% [88]. On the other hand, Chiaverini et al. (2016) demonstrated the benefit of treating a wound difficult to heal in two pediatric patients diagnosed with junctional EB applying topically 0.5% timolol maleate, leading to complete cicatrization within three weeks [89]. Timolol maleate is a β-blocker traditionally used as an ophthalmic solution to reduce intraocular pressure. When applied to wounds, timolol maleate promotes keratinocyte migration for wound healing by blocking β-2 receptors on keratinocytes, which are responsible for inhibiting this migration [89]. Overall, timolol maleate is believed to have a favorable safety profile with no serious adverse effects. The typical treatment regimen is one drop applied 2–3 times daily [90]. Timolol maleate can be compounded in concentrations of 0.25% to 0.5% in gel, cream, or solution forms [91,92,93].

**Table 2:** The main topical treatments for epidermolysis bullosa (EB) reported in the literature.

Study	Treatment	Drug Concentration (*w*/*w*%)	Pharmaceutical Form	Posology	Main Findings
Schwieger-Briel et al. [64] Kern et al. [65] Murell et al. [66]	Commercial medicine (Filsuvez^®^)	10% of the triterpene dry extract from Betulae bark (birch bark)	Oleogel	The medicine was applied every 24 to 48 h for 14 days [64], applied once every 4 days for 45 days [65] or 24 months [66].	Trend toward faster wound epithelialization with Oleogel-S10 versus the vehicle in DEB patients, with good tolerability and patient-reported efficacy * [64]; Significant improvement of wound closure rates by day 45 compared to control gel, mainly in patients with recessive dystrophic EB; AE’s: mostly mild/moderate; one serious AE Oleogel-S10 related [65]; Sustained statistically significant reduction in wound burden over 24 months [66]
Paller at al. [71]	Investigational medicine (SD-101)	6% Allantoin	Cream	Cream was applied once a day for 3 months	No difference between SD-101 and vehicle for complete wound closure or time to closure; trend toward faster closure in children 2–< 12 years and wounds ≥ 5%; well tolerated [71].
Guttmann-Gruber et al. [75]	Compounded medicine (Psorcutan^®^ or Daivonex^®^ dilution in Ultraphil^®^ base)	0.0005% Calcipotriol	Ointment	Daily topical application for 4 weeks	Significant wound area (day 14) and pruritus (day 14/28) reduction versus placebo; no drug-related AEs or serum calcium changes; Improved wound microbiome *.
Wally et al. [78]	Investigational medicine (AC-203)	1% Diacerein	Cream	Once daily for 4 weeks	Statistically significant reduction in blister number versus placebo; significant absolute blister reduction after follow-up; no AEs.
Chelliah et al. [82]	Commercial medicine	Canabidiol	Oil solution	Two or three times per day	Fewer blisters and faster healing of active blisters; no self or family-reported AEs from topical CBD **
Yasar et al. [87]	Compounded medicine	10% Sucralfate	Cream	Once a day for 2 weeks	On day 10, improved lesions on the genitalia and lower extremities) **
Chiaverini et al. [89]	Commercial medicine (Timoglau ^®^)	0.5% Timolol maleate	Solution	One droplet, 2–3 times daily, for 3 weeks	After 3–8 weeks twice daily application of 0.5% timolol maleate eye drops led weeks 100% and 80% wound healing in patients 1 and 2, respectively **

EB: Epidermolysis bullosa; AEs: Adverse Effects; * Results without statistical significance; ** Without statistical analysis.

### 2.3. Topical Treatment of Hailey–Hailey Disease (HHD)

The topical treatment of HHD aims to control inflammation, prevent secondary infections, and reduce discomfort [94]. However, its management remains a challenge due to the chronic and recurrent nature of the condition, the predisposition of lesions to worsen with heat, moisture, and friction, and the limited availability of curative therapies. Adjunctive measures, such as minimizing exposure to heat and humidity, reducing mechanical friction, and maintaining proper skin hygiene, are essential to prevent recurrences and enhance the effectiveness of topical agents [95]. Therefore, Bittencourt et al. (2024) [96] described the case of a 55-year-old female patient with HHD managed with topical 15% aluminum chloride in aqueous solution (roll-on), applied exclusively to the affected areas, biweekly. After eight weeks of treatment, the patient showed near-complete remission of the lesions with an improvement in quality of life [96]. The 15% aluminum chloride solution (roll-on) proved effective in improving HHD, which may be attributed to its potent antiperspirant and astringent properties that reduce moisture and friction, two key factors that exacerbate the cutaneous manifestations often associated with HHD [96].

On the other hand, to manage inflammation, Khang et al. (2023) [97] reported the case of a 53-year-old woman with HHD treated with topical ruxolitinib cream 1.5%, twice daily, which led to a significant improvement of residual lesions within just one day, and complete resolution after one month of treatment. The fact that Janus Kinase (JAK) inhibitors represent a novel class of targeted therapies with potential applications in various inflammatory dermatoses underscores its potential relevance in treating refractory HHD. These target agents act by inhibiting the JAK-STAT signaling pathway, which is involved in the activity of numerous cytokines, including interferon alpha/beta, interferon gamma, and IL 2, 4, 5, 6, 12, 13, 15, and 23 [97].

### 2.4. Topical Treatment of Darier Disease

The topical treatment of DD aims to reduce inflammation, hyperkeratosis, and secondary infections. Despite the symptomatic relief provided by these therapies, DD remains a chronic condition that requires individualized management and long-term care [9]. Treatment remains challenging due to the chronic and recurrent nature of the condition, which is often exacerbated by heat, sweating, UV exposure, and mechanical friction. Additionally, secondary bacterial or viral infections are common and may further complicate clinical course. Although no topical therapies are currently approved specifically for DD, several commercially available agents are used off label to manage localized or mild forms (Table 3) [9,25].

To address persistent inflammation often associated do this disease, Campos et al. (2023) [98] reported the topical application of a gel containing 3% sodium diclofenac and 2.5% hyaluronic acid in a natrosol base, twice daily for eight weeks, on a 32-year-old male patient diagnosed with DD. After four months of continuous use a significant regression of cutaneous lesions was achieved, with no local or systemic adverse effects or significant lesion recurrence [98]. Santos-Alarcón et al. (2016) [99] reported similar findings in a 33-year-old patient with treatment-resistant DD, successfully managed with topical 3% sodium diclofenac and 2.5% hyaluronic acid, applied once daily to the anterior chest and abdomen. After six months of continuous use, significant clinical improvement was observed, including flattening of the lesions, reduced roughness, and partial disappearance of the papules, without local or systemic adverse effects [99]. Millán-Parrilla et al. (2014) also reported benefits from the application of 3% sodium diclofenac gel in two patients with DD [100]. The combination of 3% sodium diclofenac with 2.5% hyaluronic acid has proven effective in the treatment of DD due to its anti-inflammatory, antiproliferative, and moisturizing properties. Diclofenac inhibits the COX-2 enzyme, reducing local inflammation and contributing to the regression of hyperkeratotic papules, while hyaluronic acid acts as a vehicle that enhances drug penetration, maintains skin hydration, and supports the repair of the skin barrier. This topical combination has been well tolerated and may represent a safe and effective therapeutic alternative for refractory cases [98,99,100].

Other off-label topical therapies that, besides targeting beyond inflammation, also aim to restore epidermal differentiation and barrier integrity DD, have been explored. Hagino et al. (2022) [101] reported the case of a Japanese patient with DD presenting with lesions on the scalp, neck, and groin, who was successfully treated with a combined ointment containing calcipotriol (50 μg/g) and betamethasone dipropionate (0.5 mg/g). Clinical improvement may be attributed to complementary mechanisms: calcipotriol promotes keratinocyte differentiation and protects against apoptosis, and betamethasone may restore SERCA2 expression by alleviating endoplasmic reticulum stress through inhibition of pro-inflammatory cytokines such as IL-6. Additionally, the formulation vehicles containing petrolatum, paraffin, and polyoxypropylene stearyl ether, may contribute to the reduction in transepidermal water loss (TEWL) through the formation of a semi-occlusive film [101].

However, Michelle et al. (2020) [102] described a 49-year-old woman with DD whose condition, previously managed with clobetasol propionate 0.05% (topical solution) and betamethasone dipropionate 0.05% (topical ointment), worsened, presenting widespread erythematous, scaly, and greasy papules, associated with an unpleasant odor. To target bad odor reduction, topical metronidazole 1% gel was additionally prescribed once daily to the affected areas. After two months of treatment, although the cutaneous lesions persisted, the patient reported complete resolution of the odor, which reappeared the following day when metronidazole application was discontinued. This observation, combined with the fact that the treatment was well tolerated, with no reported adverse effects, suggests that 1% metronidazole gel may be a safe and effective option for odor control in patients with DD [102]. This efficacy is due to its potent antimicrobial and antiseptic properties. The unpleasant odor commonly associated with DD lesions is often attributed to bacterial overgrowth, particularly of anaerobic microorganisms growing on inflamed and macerated skin. By effectively reducing the microbial load, metronidazole can significantly reduce or even eliminate the smell [102].

**Table 3:** The main topical treatments for Darier Disease (DD) reported in the literature.

Study	Treatment	Drug Concentration (*w*/*w*%)	Pharmaceutical Form	Posology	Main Findings
Campos et al. [98]; Santos-Alarcon et al. [99]; Millán-Parrilla et al. [100]	Commercial medicine (Solaraze^®^)	3% sodium diclofenac and 2.5% hyaluronic acid	Gel	Twice daily for 8 weeks [98] Once daily for 6 months [99] Twice a day for 3 (patient 1) and 5 (patient 2) months, respectively [90]	After 4 months, lesion regression with residual macules and few keratotic papules was observed (photographic records), which maintained after discontinuation ** [98]; After six months, hyperkeratotic papules were flattened without local AEs ** [99]; After treatment’s duration, DD lesions were resolved, with only patient 2 reporting irritation on the neck after application ** [100].
Hagino et al. [101]	Commercial medicine (Dovobet^®^)	0.005% calcipotriol and 0.05% betamethasone dipropionate	Ointment	Once a day for 3 months	After three months, there was a reduction in redness and scalp scaling, with flattened papules on the neck and groin; only mild remaining pigmentation.
Michelle et al. [102]	Commercial medicine	0.005% Clobetasol propionate; 0.05% betamethasone dipropionate; 1% metronidazole	Solution, ointment and gel, respectively	Once daily for 2 months	Topical metronidazole 1% gel was well tolerated and with no AEs reported. It resolved malodor within 2 months despite persistent lesions; malodor recurred if treatment was stopped **.

DD: Darier Disease; AEs: Adverse Effects; ** Without statistical analysis performed.

### 2.5. Topical Treatment for Congenital Ichthyosis

Conventional treatments for ichthyosis focus on symptom relief as the first approach, since they are available, economical, and well-tolerated. These treatments range from mechanical scale removal to systemic pharmacotherapy, aiming at immediate symptom relief and long-term disease control [31], which may be sufficient in mild cases [103]. Due to the chronicity and widespread skin involvement of the disease, graft contraction commonly occurs, and the need for repeated surgeries are common, thus positioning topical therapy as the most appropriate long-term approach [104]. Despite the lack of approved therapies for congenital ichthyosis, several topical strategies have been explored, aiming to restore epidermal function (Table 4).

Hyperkeratosis is one of the main symptoms of ichthyosis, and its management with keratolytic agents to promote epidermal turnover and reduce scale accumulation has been demonstrated to be effective in a few studies [10]. In a phase II trial, Paller et al. (2022) demonstrated that topical isotretinoin 0.1% was more effective than 0.2% in treating various forms of congenital ichthyosis over eight weeks, supporting its potential as a topical alternative to systemic retinoids [105]. Also, Craiglow et al. (2013) reported the case of a 77-year-old woman with recessive ichthyosis and long-standing keratinization disorders, that was managed with 0.1% tazarotene cream, applied daily to both lower eyelids, which improved ocular discomfort and the degree of ectropion within two weeks, without side effects [106]. Both isotretinoin and tazarotene are topical retinoids indicated for the treatment of fine lines, skin pigmentation, and roughness on the facial skin. They are also effective in treating both inflammatory and non-inflammatory lesions. The activity of retinoids is related to the inhibition of sebaceous gland development, reduction in sebaceous secretion, inhibition of follicular keratinization, and anti-inflammatory activity. Retinoids enhance epidermal turnover, thicken the epidermis, restore the granular layer, compact the SC, reduce melanin content, and may contribute to collagen synthesis in the dermis [106,107]. Both isotretinoin and tazarotene can be formulated in concentrations of 0.05% or 0.1% in gel and cream forms [108,109].

Besides the use of retinoids, other strategies have been adopted to overcome exacerbated skin thickness. Kaplan et al. (2018) showed that the topical application of N-acetylcysteine at concentrations of 5 to 10% lead to an improvement of hyperkeratotic lesions in patients with ichthyosis within two weeks, with only a few patients reporting irritation, erythema, and intolerance to the medicinal odor [110]. Topical N-acetylcysteine can inhibit keratinocyte proliferation, exerting a therapeutic effect primarily on hyperproliferative skin diseases without cytotoxic effects [111]. Its main disadvantage is the unpleasant odor, caused by the progressive oxidation of N-acetylcysteine and consequent release of sulfur-containing components [12]. To improve application and eliminate the intense sulfur odor, Davila-Seijo et al. (2014) reported a case of hereditary lamellar ichthyosis successfully treated with a well-tolerated combination of 10% N-acetylcysteine cream, urea cream, and a 1.5% rosemary oil solution [112]. Furthermore, González-Freire et al. (2022) developed another strategy to overcome the strong odor: a cream combining 10% carbocisteine and 5% urea, which exhibited good tolerability and no reported adverse effects in patients with ichthyosis [113]. Carbocisteine differs from N-acetylcysteine by lacking a free –SH group, which leads to improved oxidative stability and prevents the release of an unpleasant odor [113]. The usual concentration for preparing N-acetylcysteine and carbocisteine in cream is 10% [114]. When combined with urea, which is moisturizing at concentrations up to 10% and keratolytic between 10% and 40%, a synergistic effect is achieved, improving both keratolytic efficacy and active ingredient penetration [114].

In recent years, there has been increasing interest in topical therapies that target the underlying pathophysiological mechanisms of ichthyosis, particularly in the development of compounded formulations for rare subtypes such as CHILD syndrome. Bajawi et al. (2018) reported the case of a one-month-old neonate diagnosed with CHILD syndrome, whose skin lesions significantly improved with the topical application of 2% simvastatin ointment, twice daily [115]. Yu et al. (2018) reported a rare and typical case of CHILD syndrome associated with multiple verruciform xanthoma (VX)-like lesions that were successfully treated with a cream that, besides containing 2% simvastatin, also included 2% cholesterol [116]. Significant improvement was reported by Sandoval et al. (2019) in a patient with CHILD syndrome treated with a topical lotion containing 2% lovastatin and 2% cholesterol, applied twice daily [117]. CHILD syndrome results from mutations in the *NAD(P)H* gene, which not only impair proper cholesterol synthesis but also lead to the accumulation of toxic sterols [117,118], justifying that cholesterol supplementation alone may be insufficient [118]. Therefore, the combination of cholesterol with statins, either simvastatin or lovastatin (potent inhibitors of HMG CoA reductase, a rate limiting enzyme of the endogenous cholesterol synthesis) may be more effective. Nevertheless, further research on lipid metabolism in the skin is needed to develop more effective treatments for CHILD syndrome and other keratinization disorders [117]. Both lovastatin and simvastatin are typically prepared at 2% in creams or gels, and cholesterol is used as an emulsifying and emollient agent in creams at a concentration of 3% [119].

All this knowledge has potentiated the development of comprehensive topical strategies that combine keratolytic, emollient, and pathophysiology-directed agents to address multiple aspects of ichthyosis in a single regimen, especially for more severe cases. Khalil et al. (2018) [120] conducted a study on 15 cases of resistant congenital ichthyosis where glycolic acid was applied to the face and the rest of the body in a concentration of 5 and 10%, respectively, and a cream combining 2% of lovastatin and 2% of cholesterol was applied every morning to all affected areas. Clinical responses improved over time, with most patients reaching very good to excellent outcomes by month three. Mild adverse effects, including erythema, peeling, and burning, were self-limited and managed by modifying application frequency [120]. Bergqvist et al. (2018) [121] also managed two cases of symptomatic CHILD syndrome using a cream containing 2% cholesterol and 2% lovastatin, with or without glycolic acid, which showed improvement in skin lesions within the first 4 weeks of treatment. The addition of glycolic acid enhanced the penetration of the cholesterol-lovastatin cream into the keratotic scales [120,121]. This study confirmed the efficacy of the pathogenesis-targeted therapy and suggested the possibility of modifying its formulation by including glycolic acid to optimize therapeutic outcomes [121].

**Table 4:** The main topical treatments for congenital ichthyosis are reported in the literature.

Study	Treatment	Drug Concentration (*w*/*w*%)	Pharmaceutical Form	Posology	Main Findings
Paller et al. [105]	Investigational medicine (PAT-001)	0.1% isotretinoin	Ointment	Twice a day for 8 weeks	0.1% isotretinoin was more effective, than 0.2%, with mild and transient local AEs *
Craiglow et al. [106]	Commercial medicine (Tazarene^TM^)	0.1% tazarotene	Cream	Once a day for 2 weeks	Rapid improvement of ocular discomfort and the degree of ectropion, without reported AEs **
Bajawi et al. [115]	Compounded medicine	2% simvastatin	Ointment	Twice a day for 2 weeks After 6 weeks, one application per day	Improvement of skin lesions, hyperkeratosis, erythema and scaling reduction in neonate with CHILD syndrome, after 2 weeks and a complete resolution after 6 weeks **
Yu et al. [116]		2% simvastatin and 2% cholesterol	Cream	Twice a day for 2 months	Improvement of skin lesions, hyperkeratotic plaques disappearance, normalization of skin texture and local inflammation reduction **
Sandoval et al. [117]		2% lovastatin –and 2% cholesterol	Cream	Three applications per week for 8 weeks.	Improvement of skin lesions, scaling, erythema and inflammation reduction, with skin texture normalization **
Khalil et al. [120]		Glycolic acid—10–20% 2% Lovastatin and 2% cholesterol	Creams	Twice a day for 3 months	Improvement of skin lesions, hyperkeratosis and erythema reduction. An average reduction of 57.5% in disease severity, after 3 months *
Bergqvist et al. [121]		2% cholesterol and 2% lovastatin; 12% glycolic acid	Creams	Twice a day for 4 weeks	Improvement of skin lesions, hyperkeratosis, erythema and scaling reduction **
Kaplan et al. [110]		5 to 10% N-acetylcysteine	Cream	No data available	Improvement of hyperkeratotic lesions, with scaling and skin stiffness reduction. Mild irritation and local erythema appeared in some patients **
Davila-Seijo et al. [112]		10% N-acetylcysteine and 10% urea	Cream	Twice a day for 6 weeks	Hyperkeratosis, scaling and skin thickening reduction, in a LI patient **
González-Freire et al. [113]		10% Carbocisteine and 5% urea	Cream	No data available	Skin texture and hydration improvement, with good tolerability and no AEs reported **

CHILD syndrome: Congenital Hemidysplasia with Ichthyosiform Erythroderma and Limb Defects syndrome; LI: Lamellar Ichthyosis; PAT-001: isotretinoin-based ointment; AEs: Adverse Effects; * Results without statistical significance; ** Without statistical analysis performed.

### 2.6. Topical Treatment for Erythrokeratoderma

Erythrokeratoderma’s manifestation begins in childhood, and it follows a chronic course, with significant impact on patients’ quality of life. Treatment is challenging and aims to manage the symptoms, as there is no definitive cure. The main topical therapeutic approaches include the use of emollients and keratolytic, such as urea, salicylic acid, and lactic acid, which promote skin hydration and help reduce hyperkeratosis, respectively. For instance, Guaraldi et al. (2013) [122] reported the case of a 55-year-old patient with progressive symmetrical erythrokeratodermia, presenting lesions on the hands, interphalangeal pads, wrists, groin, and feet. The patient was treated with topical 0.025% tretinoin combined with 20% urea and 3% salicylic acid, in addition to a moisturizing lotion applied once a day in the morning. After four months of treatment, only mild clinical improvement was observed [122].

Both topical retinoids, such as tretinoin and adapalene, which enhance cell turnover and help reduce skin thickening, and topical corticosteroids, used during exacerbations to reduce inflammation and erythema, can be used to manage erythrokeratoderma. Calcineurin inhibitors, such as tacrolimus, can also be useful, especially in sensitive areas, such as the face or intertriginous regions, due to their lower risk of inducing skin atrophy compared to corticosteroids [122,123].

However, Tarikci et al. (2016) [123] reported the case of a 7-year-old child diagnosed with PSEK, presenting erythematous plaques on the face and elbows, that was successfully treated with calcipotriol ointment twice daily, after one month. No topical corticosteroids or adjuvant emollients were used during the treatment [123].

### 2.7. Topical Treatment for Porokeratosis

Porokeratosis is a genodermatosis characterized by disorders in keratinization and the presence of the cornoid lamella, with a potential risk of malignant transformation. Consequently, treatment remains a challenge, especially in extensive or refractory forms [12]. There are no approved therapies for porokeratosis. Nonetheless, there are several reports of commercially available topical treatments used off label to manage porokeratosis, as well as some drugs not even indicated for topical application (Table 5).

As hyperkeratosis is a common feature of porokeratosis, the use of keratolytic agents, namely retinoids and hydroxy acids, may be beneficial in managing epidermal thickening. In fact, Severson et al. (2024) reported a marked improvement of a 74-year-old man with diagnosed with DSAP using 0.05% tretinoin cream combined with 0.005% calcipotriene cream and the daily use of moisturizers to prevent skin irritation [124]. Tretinoin and calcipotriene act complementarily in DSAP, while tretinoin promotes epidermal renewal and reduces the formation of the cornoid lamella, calcipotriene regulates gene expression and has anti-inflammatory properties [124]. Alomran & Kanitakis (2020) [125] also reported a marked improvement in a 38-year-old woman with porokeratotic eccrine ostial and dermal duct nevus (PEODDN), who was treated with topical tazarotene 0.1% gel. This treatment`s effectiveness had been previously exhibited for different keratinization disorders, both genetic and acquired [125]. Moreover, Lang et al. (2020) [126] reported a series of five female patients with a history of DSAP who were successfully treated with 50% glycolic acid and 25% salicylic acid using a two-layer application technique. This resulted in a clear reduction in lesions in 3 sessions, with low incidence of side effects [126]. Chemical peeling using glycolic (an alpha-hydroxy acid) and salicylic acid (a beta-hydroxy acid) promotes the elimination of the cornoid lamella, characteristic structure of DSAP composed of parakeratotic cells, while stimulating the proliferation of new keratinocytes, favoring the replacement of altered cells with normal ones. Salicylic acid also has anti-inflammatory properties, contributing to the reduction in erythema and local irritation [126].

More recently, topical strategies combining cytotoxic and immunomodulatory activities have emerged as promising strategies to enhance therapeutic outcomes in porokeratosis. Portelli et al. (2024) described the case of a 29-year-old patient with an irregular porokeratosis plaque on the right hand that was successfully treated with 5% 5-fluorouracil (5-FU) cream [127]. Furthermore, Yeh et al. (2021) [128] reported the case of a 46-year-old man with porokeratosis treated with a combined regimen of 5% of 5-FU cream in a 1:1 weight ratio with 0.005% calcipotriene cream. This resulted in complete clearance of the lesions, with no recurrence observed for 28 months [128]. The combination of 5-FU and calcipotriene acts on porokeratosis through a synergistic mechanism involving the induction of an adaptive immune response: 5-FU promotes the death of abnormal keratinocytes by inhibiting deoxyribonucleic acid (DNA) synthesis, while calcipotriene stimulates the expression of the cytokine Thymic Stromal Lymphopoietin, activating dendritic cells that, in turn, trigger the activation of CD4^+^ T cells. This response results in localized lesion clearance and the induction of cutaneous immune memory, contributing to sustained remission and the potential prevention of recurrences or malignant transformation [127,128].

Topical therapies targeting the underlying metabolic and keratinization abnormalities in porokeratosis are gaining attention in current dermatological research. Both Santa Lucia et al. (2024) and Diep et al. (2023) reported that topical formulations containing 2% lovastatin, with or without cholesterol, effectively reduced disease severity in DSAP and bilateral linear porokeratosis, with no significant adverse events [129,130]. Cases of linear porokeratosis, either standalone or coexisting with CHILD syndrome diagnoses, DSAP, and disseminated palmar and plantar porokeratosis were successfully treated with the same cholesterol/lovastatin combination cream, with no adverse side effects reported [119,131,132,133]. In addition, Wozna et al. (2024) reported a case of a refractory Mibelli-type porokeratosis lesion, unresponsive to corticosteroids, antibiotics, and tacrolimus, that showed marked reduction in size and improvement in the appearance of the lesion after 9 months of treatment with 2% lovastatin/2% cholesterol ointment [51]. However, both Atzmony et al. (2021) and Ugwu et al. (2020) reported that cholesterol 2% monotherapy for porokeratosis lesions resulted in no clinical improvement [119,133]. A different statin was used by Raison-Peyron et al. (2025) [134], who successfully treated a case of linear porokeratosis on the lower limb in a 74-year-old patient with twice-daily topical 2% simvastatin/2% cholesterol in a cold cream base (paraffinum liquidum, cera alba, cetyl palmitate, and water). However, lesion recurrence occurred shortly after switching to a compounded preparation with a different cream base (cetyl alcohol, propylene glycol, pentylene glycol, and chlorphenesin), underscoring the importance of vehicle composition in topical therapy [134]. Statins, namely lovastatin, atorvastatin and simvastatin, exert a therapeutic effect in DSAP by inhibiting the enzyme HMG-CoA reductase, a key component of the mevalonate pathway. This inhibition prevents the accumulation of toxic intermediate metabolites, which may help avoid the excessive activation of the local immune response, considered as one of the causes of the characteristic phenotypic presentation of DSAP. However, since cholesterol is one of the main lipidic components of the skin barrier, including it in the formulation combined with statins is crucial to restore skin cholesterol levels, in addition to the blockage of the cholesterol biosynthetic pathway [119,129,130,131,132,133].

**Table 5:** The main topical treatments for Porokeratosis reported in the literature.

Study	Treatment	Drug Concentration (*w*/*w*%)	Pharmaceutical Form	Posology	Main Findings
Santa Lucia et al. [129] Chen et al. [131] Atzmony et al. [119]	Compounded medicines	2% lovastatin and 2% cholesterol Cream	Cream	Once or twice a day for 3 months [129] Twice a day for 4 weeks [131] Twice a day for 3 months [119]	In DSAP reduced clinical severity and inflammation [129]; In linear and disseminated PK reduced the number and size of lesions [131]; In palmar and plantar PK decreased thickness [119] **
Wozna et al. [51] Diep et al. [130] Ugwu et al. [133]		2% lovastatin and 2% cholesterol	Ointment	9 months [51] Twice a day for 3 months [130] Twice a day for 6 weeks [133]	Plaque size and scaling reduced [51,130,133]; Progressive reduction in diameter and esthetic improvement of refractory Mibelli-type lesions [51]; Complete resolution of lesions [133]. Effective and sustained response in linear PK, with no recurrence [130,133] **
Sultan et al. [132]		2% lovastatin and 2% cholesterol	Gel	Once a day for 2 months	Reduction in hyperkeratosis, erythema and scaling in patients with linear Porokeratosis, DPSA and disseminated palmar and plantar PK. No local and systemic AEs were observed **
Raison-Peyron et al. [134]		2% simvastatin and 2% cholesterol	Cream	Twice a day for 2 months	Reduction in skin lesion thickness, erythema and scaling, in a patient with linear PK **
Severson et al. [124]	Commercial medicine (Ketrel^®^ and Daivonex^®^)	0.05% tretinoin. 0.005% calcipotriene	Cream	Applications at night, once every 2 days, for 6 months	Reduction in number and size of DSAP lesions and enhancement of skin texture **
Portelli et al. [127]	Commercial medicine (Efudix^®^)	5% of 5-FU	Cream	30 mg daily for 7 weeks	Total resolution and no lesions recurrence in patients with irregular PK plaque. Mild and transient AEs were reported **
Yeh et al. [128]	Compounded medicine	5% of 5-FU and 0.005% of calcipotriene	Cream	Twice a day for 5 days	Complete clearance of the lesions, with no recurrence observed for 28 months **
Lang et al. [126]	Compounded medicine	50% glycolic acid and 25% salicylic acid	Solution	Three peeling cycles repeated every 6 weeks	Reduction in the number and size of DSAP lesions in five patients, after 3 peeling sessions, with skin texture and pigmentation improvement and low AEs **
Alomran & Kanitakis [125]	Commercial medicine (Tazarene^TM^)	0.1% tazarotene	Gel	Daily application for 1 month	Reduction in skin lesion thickness and scaling in a PK eccrine ostial and dermal duct nervus case, with mild local irritation **

PK: Porokeratosis; 5-FU—5-fluorouracil; AEs: Adverse Effects; ** Without statistical analysis.

### 2.8. Topical Treatment of Inflammatory Linear Verrucous Epidermal Nevus

Treatment of ILVEN is challenging and aims to reduce pruritus, improve skin appearance, and enhance the patient’s quality of life. Currently, there are no effective therapies for all ILVEN cases, as success depends on the patient’s age, as well as the location and size of the lesions [135,136].

Most conventional management strategies include topical corticosteroids, calcineurin inhibitors, keratolytic agents, and retinoids, which may help to alleviate inflammation and hyperkeratosis (Table 6). For example, Wollina et al. (2017) reported the case of an 18-month-old female patient with ILVEN who did not respond to topical tacrolimus but achieved good results with topical application of 0.1% mometasone furoate, a topical corticosteroid widely used to treat inflammatory and allergic skin conditions, under occlusion for 2 weeks, with no signs of recurrence [137,138]. Mometasone is generally available as mometasone furoate at a concentration of 0.1% and can be compounded in pharmaceutical forms such as ointments, creams, lotions, and topical solutions, at concentrations of up to 1.0% [138]. Subsequently, Zemero et al. (2021) reported that a treatment using hydrocortisone, desonide, clobetasol propionate, also topical corticosteroids with anti-inflammatory, antipruritic, and vasoconstrictive effects, combined with 3% salicylic acid, and 5% dexpanthenol resulted in a reduction in keratosis, inflammatory signs, and symptom control in patients with ILVEN [139]. Topical corticosteroids primarily target fibroblasts and keratinocytes located between the dermis and epidermis and bind to specific regions that regulate the inflammatory process, inhibiting the release of IL-1, IL-2, IL-6, interferon, tumor necrosis factor, and T-cell proliferation, resulting in anti-inflammatory, antiproliferative effects and reduced erythema [140]. Hydrocortisone can be compounded in concentrations ranging from 0.1% to 2.5%; desonide can be compounded at 0.05% to 0.1%; and clobetasol propionate is usually compounded at a concentration of 0.05% [140]. Topically applied salicylic acid, included in this topical therapy for its skin exfoliating, bacteriostatic and fungicidal properties in concentrations above 2%, can be compounded in concentrations up to 40% [141]. The concomitant incorporation of ingredients such as dexpanthenol, which provides emolliency and enhances the hydration of xerotic and rough skin, may be compounded at concentrations between 0.5% and 5%, depending on the intended therapeutic or cosmetic purpose [142].

When corticosteroids and keratolytics fail to control marked hyperkeratosis and persistent scaling related to ILVEN, cytotoxic agents may be an alternative to directly target the abnormal keratinocyte differentiation. Even though Bien et al. (2023), shown that treating a patient with severe ILVEN was with a topical solution containing 0.5% 5-FU (5-Fluorouracil) and 10% salicylic acid twice daily to two selected areas, proved to be less effective than cryosurgery [135], Al Abadie et al. (2018) reported that 5% 5-FU cream applied either daily for 2 weeks or in two 3-week cycles separated by a 1-month break resulted in an 80–90% improvement in lesions [143]. 5-FU is a cytotoxic, immunosuppressive pyrimidine analog that inhibits thymidylate synthase, a key enzyme in pyrimidine synthesis, ultimately impairing DNA and RNA production and leading to selective damage of rapidly proliferating cells [135]. It can be compounded at concentrations ranging from 0.5% to 5% in creams or solutions [137]. The combination of 5-FU and salicylic acid provides a complementary therapeutic strategy for ILVEN: salicylic acid’s keratolytic activity, in concentrations up to 10%, leads to the disruption of the hyperkeratotic barrier, thereby enhancing 5-FU penetration, while 5-FU targets the underlying keratinocyte hyperproliferation. This synergistic action addresses both the inflammatory and proliferative components of ILVEN, potentially accelerates clinical improvement, and may allow for the use of lower, less irritating concentrations of 5-FU [18,135,137].

Since inflammation and pruritus can remain despite keratolytic and cytotoxic approaches, topical anti-inflammatory agents have also been explored. Barney et al. (2019) [144] developed a 2% crisaborole ointment to treat facial lesions in a patient. After 2 months, it resulted in significant improvement, leading to the expansion of crisaborole application to all affected areas. After 3 months, all plaques became less erythematous, the lesions flattened, and pruritus disappeared. Crisaborole is a phosphodiesterase 4 (PDE4) enzyme inhibitor, which increases intracellular cyclic adenosine monophosphate (cAMP) levels, thereby reducing the production of inflammatory cytokines and chemokines such as IL-4, IL-3, and prostaglandin E2 [144].

Despite the previously described topical options that target either keratinocyte hyperproliferation or inflammation, there are immunomodulatory agents that may combine both effects, resulting in a potentially more comprehensive approach. Building upon this, Patel et al. (2020) [145] described the off-label use of topical sirolimus at a concentration of 0.4%, in a petroleum jelly ointment base, to be applied once at night. The patient experienced significant improvement in both the pruritus and the skin lesion within four weeks, with persistent improvement [145]. Sirolimus is an inhibitor of the mammalian target of rapamycin (mTOR), possessing antiproliferative, antiangiogenic, and immunosuppressive properties. It downregulates T-cell proliferation and, consequently, the production of IL-1, IL-6, and TNF-α, which makes it a suitable treatment option for ILVEN [146]. Sirolimus or Rapamycin was isolated from the bacterium *Streptomyces hygroscopicus* and can be compounded as a gel, cream, or ointment at a concentration of 0.2%, and should be stored under refrigeration [145,147].

**Table 6:** The main topical treatments for ILVEN reported in the literature.

Study	Treatment	Drug Concentrations (*w*/*w*%)	Pharmaceutical Form	Posology	Main Findings
Bien et al. [135]	Commercial medicine (Actikerall^®^)	0.5% 5-FU 10% salicylic acid	Solution	Once a day for 8 weeks	Partial improvement of erythema and scaling, but less effective than cryosurgery in patients with severe ILVEN **
Al Abadie et al. [143]	Commercial medicine (Efflurak^®^)	5% 5-FU	Cream	Once daily for one week per month in pulsed cycles for 3 months	ILVEN lesions improved from 80% to 90%. Reduction in erythema, scaling and pruritus, with no recurrence **
Wollina et al. [137]	Commercial medicine (Elocon^®^)	0.1% mometasone furoate	Ointment	Once daily for 8 weeks	Under occlusion, resulted in erythema and scaling reduction, and almost complete lesion resolution, with no recurrence **
Zemero et al. [139]	No data available	Hydrocortisone–Desonide– Clobetasol propionate; 3% salicylic acid; 5% dexpanthenol	No data available	No data available	Reduction in keratosis, inflammation and pruritus, in patients with ILVEN **
Barney at al. [144]	Commercial medicine (Staquis^®^)	2% crisaborol	Ointment	Twice a day for 2 months	Plaques progressively improved, erythema reduction, flattening, and pruritus resolution, without AEs reported **
Patel et al. [145]	Commercial medicine (Hyftor^®^)	0.2% sirolimus	Ointment	Twice a day for 3 weeks	Reduction in pruritus and skin lesion, with tolerability. **

ILVEN: Inflamatory Linear Verrucous Epidermal Nevus; 5-FU: 5-fluorouracil; AEs: Adverse Effects; ** Without statistical analysis.

### 2.9. Topical Treatment for Piebaldism

Although the depigmented areas of piebaldism are benign and non-progressive, their appearance can be socially debilitating for some patients. Treatment is challenging and aims to improve esthetics, including skin grafting, cell transplantation, camouflage techniques using makeup, and the use of hair dye for poliosis, to enhance the quality of life of patients [56]. It is essential to protect the white patches, which lack melanocytes, from sunburn, as well as applying protective creams and gels to prevent malignant transformation [147,148].

Unlike other rare genodermatoses addressed in this review, no case reports or clinical trials specifically evaluating topical therapies for piebaldism were identified in the literature. Consequently, recommendations for photoprotective management are based on general sunscreen formulations and compounding strategies rather than disease-specific studies. As an example of photoprotective gel-creams, octyl methoxycinnamate, an UVB sunscreen filter used to stabilize and complement the action of other filters, increasing the photoprotection efficiency of the product, can be compounded in concentrations of 8 to 10% [146]. Another example is diethylamino hydroxybenzoyl hexyl benzoate (Uvinul^®^ A Plus), a UVA sunscreen filter, which can be compounded in concentrations of 4 and 5%. Bis-ethylhexyl oxyphenol methoxyphenyl triazine (Tinosorb^®^ S) is a UVA and UVB sunscreen filter and can be compounded in concentrations of 3 and 5% in a gel-cream base [149,150]. Photoprotective makeup can also be compounded by incorporating a pigment mixture at 10% in gel-cream base and glycerin at 10% as an emollient.

## 3. Discussion

Even though rare genetic dermatological diseases are frequently underdiagnosed and neglected in healthcare systems due to their low prevalence and limited clinical and therapeutic data, the increasing number of clinical studies on topical treatment efficacy reflects a promising shift towards improved management strategies. Our review identified porokeratosis, EB, and congenital ichthyosis as the rare genodermatoses with the greatest number of reported studies and topical treatment options explored. This may be attributed to their relatively higher prevalence, substantial clinical morbidity, significant esthetic impact, chronic course and, in certain subtypes, risk of malignant transformation, all of which drive both research interest and therapeutic innovation [7,12,16,30,31,46]. In contrast, dermatological conditions such as piebaldism, erythrokeratodermia, and HHD have fewer reported topical interventions. In the case of piebaldism, the absence of specific topical therapies may be related to its benign, non-progressive nature, low prevalence, and predominantly esthetic or photoprotective management [56]. Erythrokeratodermia and HHD, while clinically relevant, are even less prevalent, reducing both research attention and clinical demand [23,24,43,44].

For most rare genetic dermatological diseases, treatment primarily aims to improve quality of life and control clinical signs and symptoms, with topical therapies playing a central role. Several pharmacological classes are employed, including keratolytics (e.g., urea, hydroxyacids, such as salicylic acid, and retinoids, such as tretinoin and tazarotene), vitamin D analogs (e.g., calcipotriol), topical corticosteroids (e.g., clobetasol, betamethasone), calcineurin inhibitors (e.g., tacrolimus, pimecrolimus), and cytotoxic or antiproliferative agents (e.g., 5-fluorouracil). In the absence of approved therapies for rare genetic skin disorders, commercially available topical medicines, originally indicated for more prevalent conditions, are often used off label to address key symptoms such as hyperkeratosis, inflammation, and barrier impairment, resulting in a broader scope of available treatment strategies [151,152]. Keratolytic agents, namely hydroxyacids and retinoids (0.1% isotretinoin and 0.1% tazarotene), which are indicated to treat skin conditions such as psoriasis or acne, have been studied to promote epidermal turnover and reduce scale accumulation in diseases where hyperkeratosis is a relevant feature, such as congenital ichthyosis and porokeratosis. In addition, 5-FU, with its ability to target and promote the death of abnormal keratinocytes, has also been reported as a possible treatment not only for ILVEN, but also for porokeratosis, due to its premalignant potential. On the other hand, rare genetic dermatological conditions with secondary inflammation, such as DD, where desmosomal dysfunction compromises skin barrier integrity and predisposes to irritation and infection, have been managed with topical diclofenac 3%, despite its approval being limited to actinic keratosis [153]. In fact, topical 3% diclofenac has been used in cases of patients with intolerance or contraindication for oral retinoids. Calcipotriol, which is often used in combination with betamethasone dipropionate for plaque psoriasis management, has been explored in EB treatment for its ability to promote wound healing and reduce pruritus, also as a promising and less irritative alternative to retinoids. In DD, its benefits are associated with the normalization of keratinocyte differentiation and reduction in hyperkeratosis and inflammation [154]. Despite the increasing number of clinical studies performed for these rare dermatological conditions, most of them are case reports, so the available evidence is limited to anecdotal observations, most of which are photographic records and patient’s improvement perception, and with a very reduced number of patients, without comparison to vehicle or established standard treatments [155,156].

Currently, EB is the only rare dermatological disease among those reviewed with an approved topical treatment. Few clinical trials have assessed topical treatments such as calcipotriol ointment, 1% diacerein cream, and 6% allantoin cream. While calcipotriol and diacerein demonstrated potential in reducing wound area and blister formation, respectively, none are currently approved specifically for this indication. On the other hand, Filsuvez^®^, a topical gel containing 10% birch bark triterpenes, has demonstrated therapeutic benefit in the management of partial-thickness wounds through its anti-inflammatory and pro-reparative properties. The formulation acts by modulating local inflammation, enhancing keratinocyte migration and differentiation, and promoting re-epithelialization, thereby providing symptomatic relief rather than addressing the underlying genetic defect of EB [157]. Several clinical trials have demonstrated a modest yet statistically significant improvement in wound closure and re-epithelialization compared with the vehicle, which motivated its approval by European Medicines Agency (2022) and the U.S. Food and Drug Administration (2024). However, the clinical trials performed did not use as a comparison the standard care, that is often wound dressings [158], nor did they evaluate its long-term efficacy and patient’s quality of life impact.

Recent progress in elucidating the genetic mutations and molecular pathways involved in rare genetic dermatological disorders has transformed the therapeutic paradigm from treatments focused primarily on symptomatic relief to those targeting the fundamental pathophysiological mechanisms. As a result of this change, emerging evidence reinforces the development of topical therapeutics aimed at modulating disease-specific molecular mechanisms, indicating a promising future for precision dermatologic care. For instance, in dystrophic EB, a topical gene therapy named Vijuvek^®^, which consists of the modification of herpes simplex 1 to contain the mutated *COL7A1* gene, is now commercially available [62,159]. Although such advanced therapies hold great promises for improving clinical outcomes and potentially addressing the underlying disease mechanisms in the long term, their development is often costly and time consuming, requiring extensive preclinical research and multiple phases of clinical evaluation before approval. Alongside these advances, compounded formulations are also being increasingly recognized as valuable alternatives for the rare genetic dermatological conditions discussed herein, especially in cases where no approved or commercially available therapies exist. Although such advanced therapies represent a promise for improving clinical results and potentially addressing the underlying disease mechanisms in the long term, their development is often costly and time-consuming, requiring extensive preclinical research and multiple phases of clinical evaluation before approval. Alongside these advances, compounded formulations are also being increasingly recognized as valuable alternatives for the rare genetic dermatological conditions discussed, especially in cases where no approved or commercially available therapies exist.

In fact, as depicted in Table 2, Table 3, Table 4, Table 5 and Table 6, approximately 45% of the topical medicines reported in the literature were compounded medicines. Compounding pharmacies play a crucial role in personalizing treatment for rare dermatological conditions. They enable the preparation of tailored formulations that fulfill individual patient needs, including specific dosing adjustments, combination of API with complementary or synergistic mechanisms of action (keratolytic, antiproliferative, immunomodulatory, antibiotic), flexibility in the selection of pharmaceutical forms and allow for the exclusion of potentially allergenic excipients, such as dyes, preservatives, or fragrances, enhancing tolerability [54]. Such strategies can enhance therapeutic effects, target multiple pathophysiological aspects of the disease simultaneously, and potentially shorten treatment duration or reduce the need for higher concentrations of a single drug.

The topical combination of cholesterol and statins, although not commercially available, exemplifies how pharmaceutical compounding can offer a targeted therapeutic approach for conditions such as porokeratosis and CHILD syndrome. This strategy simultaneously aims to restore cholesterol skin levels, an essential lipid component of the skin barrier, that is deficient in these disorders and inhibit the defective cholesterol biosynthesis pathway, thereby reducing the accumulation of cytotoxic sterol intermediates in keratinocytes. Also, the topical combination of N-acetylcysteine or carbocisteine with 10% urea to manage ichthyosis illustrates the potential of repurposing mucolytic agents, which are conventionally indicated for respiratory conditions, in a dermatological context, where their ability to inhibit keratinocyte proliferation may be therapeutically beneficial. This approach not only reorients the therapeutic target but also involves a change in the route of administration, from systemic to topical. The combination with 10% urea, a keratolytic agent, besides treating hyperkeratosis, further enhances the penetration of the selected active pharmaceutical ingredient (API). For diseases with fewer therapeutic options, compounded formulations can help with therapeutic gaps. However, challenges remain, including limited clinical evidence, the absence of proven active agents, lower prescription rate due to low prevalence or a milder clinical course and potentially high cost [7,12,23,24,30,31,43,44,46,56].

Whether referring to off-label use of existing topical products or to customized compounded preparations, creams, gels, and ointments are frequently used as the main pharmaceutical forms to relieve discomfort, restore the skin barrier, reduce inflammation, prevent secondary infections, and regulate keratinocyte growth and differentiation. Studies investigating the influence of vehicle and pharmaceutical dosage form on topical treatment adherence have demonstrated that these factors significantly affect patient adherence. Specifically, in patients with psoriasis, adherence was notably higher with gels and creams compared to ointments when the affected body area was extensive. In contrast, ointments provided higher adherence when the affected area was limited. However, it is important to note that none of the studies on the different topical treatments to manage EB, DD, HHD, congenital ichthyosis, porokeratosis, piebaldism and erythrokeratodermia considered patient preferences or directly measured adherence outcomes. Therefore, to optimize treatment success, the selection of the topical dosage form should be guided not only by clinical characteristics but also by individual patient preferences, as both play a crucial role in ensuring adherence and enhancing therapeutic efficacy.

In our research, innovative delivery systems, such as nanocarriers, bioengineered membranes, and advanced wound dressings, were not found, suggesting that current therapeutic approaches for the studied condition still rely on conventional formulations.

Nevertheless, the therapeutic option selected should consider the extent of the lesions, the clinical subtype, the risk of malignancy, and the patient’s tolerability. Even though topical therapies may be effective in localized cases, some require complementary approaches such as cryotherapy, laser treatment, or surgical excision, depending on the extent of the lesions and severity [51,119,130,131,132,133,134,135]. For example, even though topical therapy is the first-line treatment for ILVEN, extensive or treatment-resistant lesions have shown favorable responses to systemic agents such as oral acitretin and TNF-α inhibitors, including etanercept and adalimumab [16,80]. For other severe cases, surgical excision remains the most successful method. Laser therapy, dermabrasion, and other surgical approaches may also be considered for refractory cases [135].

The use of moisturizing cosmetic products, some with keratolytic properties, as co-adjuvants to protect the fragile skin barrier, improve skin dryness, or to reduce irritation and scaling is frequent across conditions such as EB, DD, HDD, congenital ichthyosis, porokeratosis, piebaldism, and erythrokeratodermia. However, the chronicity of these conditions demands sustained use of topical treatments, and when added to other therapy related and medical costs, this imposes a considerable financial burden [160,161]. Even though that are some countries, such as Portugal, with specific regulatory frameworks that allow for full reimbursement of these treatments for patients with ichthyosis, through the national health service [162], the overall financial impact remains substantial in many regions. In several European countries, including Germany and the United Kingdom, studies have shown that most costs associated with EB are supported by patients and their families, particularly for non-medical expenses such as wound care supplies and informal caregiving [160]. This disparity highlights the need for harmonized reimbursement policies and broader inclusion of rare dermatological conditions in national funding strategies.

Overall, topical therapies offer great advantages, such as localized action that minimizes systemic side effects, the ability to personalize treatment to individual needs, and a favorable long-term safety profile compared to systemic treatments. These benefits are particularly relevant in chronic, early-onset diseases that require lifelong management [157]. Despite their broad use, an up-to-date review of studies describing the full range of available topical treatments, their mechanisms of action, indications, side effects, and clinical efficacy was lacking. This gap could have hindered the development of clear therapeutic guidelines and limited patient access to optimal care. Given the marked variability in disease presentation and treatment response among patients, individualized therapy remains essential for managing rare genetic dermatological diseases. Looking ahead, pharmaceutical compounding may be increasingly adopted, as it enables the incorporation of APIs with established efficacy and safety profiles while allowing formulations to be tailored to patients’ specific needs and preferences. Moreover, given the low prevalence of these rare dermatological conditions, hospitals are uniquely positioned to maintain direct communication with patients, enabling the development of individualized topical formulations, the systematic evaluation of both effectiveness and tolerability, and facilitating adjustments based on individual needs. Therefore, compounding medicines, namely using already approved drugs (repurposing strategy), may be particularly advantageous compared to conventional and advanced precision therapies, where clinical trials are often infeasible due to the rarity of these conditions, providing a flexible, cost-effective, and patient-centric approach that addresses immediate clinical needs.

## 4. Methods

### 4.1. Search Strategy

The primary research questions guiding this literature review is: “What are the topical treatment options for rare genetic cutaneous disease, such as inflammatory linear verrucous epidermal nevus (ILVEN), epidermolysis bullosa (EB), Hailey–Hailey disease (HHD), Darier disease (DD), ichthyosis, erythrokeratodermias, porokeratosis and piebald’s”.

A narrative search was conducted across the Scientific Electronic Library Online (SciELO), MEDLINE^®^/PubMed^®^, Embase and Cochrane databases, from January 2013 to June 2025. The following keywords were used in both Portuguese and English: “Topical treatment and inflammatory linear verrucous epidermal nevus (ILVEN)”, “topical treatment and epidermolysis bullosa,” “topical treatment and Hailey-Hailey disease”, “topical treatment and Darier disease”, “topical treatment and ichthyosis”, “topical treatment and erythrokeratodermias”, “topical treatment and porokeratosis”, “topical treatment and piebaldism,” including their combinations.

The analysis was carried out by sequentially screening titles, abstracts, and finally, the full-text reading of the selected studies. Studies providing relevant information to answer the question were selected for inclusion: “What are the topical treatments for rare genetic dermatological diseases?”.

### 4.2. Eligibility Criteria

The inclusion criteria for study selection were (a) articles published in Portuguese or English, available in full, some of which were freely accessible; (b) original and clinical studies (randomized controlled trials—RCTs; observational studies; case series; and case reports) were included; (c) literature that discussed topical treatment of rare dermatological diseases and the contribution of personalized formulations; (d) Studies that employed topical treatment exclusively.

Exclusion criteria were applied as follows: (a) publications prior to 2013; (b) studies not related to the subject matter; (c) opinion articles, editorials, letters to the editor, and conference abstracts without complete data; (d) preclinical studies (in vitro and/or in animal models); (e) articles published in other languages, such as Spanish and French; (f) studies that employed both topical and systemic treatment; (g) articles outside the defined scope.

### 4.3. Quality Assessment

The methodological quality of the studies included was independently assessed by two reviewers. This assessment utilized predefined criteria, which included: clarity of the objectives and study design, description of topical treatment protocols, reported outcomes, and discissions based on literature. Discrepancies between the reviewers were solved through discussion and consensus. Moreover, studies with inconsistent data, unclear methodology or insufficient results description were excluded.

## 5. Conclusions

This review provides a comprehensive overview of topical treatment strategies for the main rare genetic cutaneous diseases, highlighting those involving pharmaceutical compounding. The analysis revealed that porokeratosis, EB, and congenital ichthyosis are the rare genodermatoses with the greatest number of reported studies and topical treatment options explored, whereas substantial gaps still exist in evidence-based therapies for many other disorders, such as piebaldism, erythrokeratodermia, and Hailey–Hailey. These gaps are often linked to the scarcity of randomized clinical trials, which may be due to the heterogeneity of disease manifestations and the logistical and regulatory barriers to conducting large-scale studies in rare disease populations. Generating robust clinical evidence through well-designed multicenter studies involving patients with heterogeneous disease presentations and different lesion distributions is critical to substantiate the therapeutic efficacy and safety of topical formulations in these rare dermatological disorders. Also, comparison with the respective vehicles/bases or with the standard care is needed to validate the efficacy of the pharmacological treatment. Overall, the body of evidence for most of the topical treatments described is fragmented, and treatment choices often rely on empirical use rather than robust comparative data.

Compounding medicines emerge as a crucial therapeutic resource in this context, not only for adjusting API dosage but also for enabling the combination of agents with synergistic mechanisms, modifying pharmaceutical forms to improve adherence, and customizing excipients to enhance tolerability. Such versatility is particularly relevant for managing chronic symptoms and improving patients’ quality of life, especially when no approved topical drugs are available. Therefore, collaboration between dermatologists and compounding pharmacists can be especially valuable in this regard, enabling the customization of formulations that align with both therapeutic goals and patient expectations, ultimately enhancing adherence and treatment efficacy.

Conventional dosage forms were used across the studies, with emphasis on creams and ointments. Exploring innovative delivery systems may also be a promising pathway to optimize therapeutic outcomes by improving drug permeation in a controlled and sustained manner, targeting release into the active site while ensuring the drug stability.

Through the compilation and critical analysis of the topical therapeutic landscape to manage EB, ichthyosis, HHD, DD, erythrokeratodermias, porokeratosis, ILVEN, and piebaldism, this review recognizes compounded medicines as key tools in addressing unmet clinical needs, underscoring the importance of interdisciplinary collaboration to foster the development of safe, effective, and individualized topical therapies for rare genetic cutaneous diseases. The compilation and organization of the available clinical information allow clinicians to identify which topical therapies have been most used, understand the rationale behind these choices, and observe efficacy outcomes and any reported side effects. In addition, the description of the posology for each topical treatment studied may offer practical value for prescribers. Although the available data is largely empirical and not always supported by robust clinical evidence, it still provides a useful reference for therapeutic decision-making in complex or under-researched cases. Additionally, the analysis of the pharmaceutical dosage forms selected, along with insights into their composition and the concentrations of each excipient used can support compounding pharmacists in formulating evidence-based preparations tailored to individual patient needs. Overall, this insight provides a valuable resource for both prescribers and compounding pharmacists, encouraging patient-centric approaches for patients with a rare skin disease, for whom therapeutic options are currently limited.

## Figures and Tables

**Table 1:** Number of articles found in each database according to the keyword used.

Descriptors or Keywords	PubMed	SciELO	Embase	Cochrane	Total
Topical treatment and inflammatory linear verrucous epidermal nevus	3	0	27	1	31
Topical treatment and epidermolysis bullosa	32	1	309	55	397
Topical treatment and Hailey–Hailey disease	15	0	1	0	16
Topical treatment and Darier disease	2	0	61	0	63
Topical treatment and ichthyosis	28	2	406	17	453
Topical treatment and erythrokeratodermias	0	0	8	8	8
Topical treatment and porokeratosis	26	0	58	4	88
Topical treatment and piebaldism	0	1	11	3	14

## Data Availability

No new data were created or analyzed in this study. Data sharing is not applicable.

## Abbreviations

The following abbreviations are used in this manuscript:

5-FU	5-Fluorouracil
ARCI	Autosomal recessive congenital ichthyosis
CBD	Cannabidiol
CHILD Syndrome	Congenital hemidysplasia with ichthyosiform erythroderma and limb defects syndrome
DD	Darier disease
DSAP	Disseminated superficial actinic porokeratosis
EB	Epidermolysis bullosa
EKV	Erythrokeratoderma Variabilis
FDPS	Farnesyl diphosphate synthase
FLG	Filaggrin
HI	Harlequin ichthyosis
HHD	Hailey–Hailey disease
HMG CoA	3-hydroxy-3-methylglutaryl coenzyme A
IV	Ichthyosis vulgaris
ILVEN	Inflammatory linear verrucous epidermal nevus
LI	Lamellar ichthyosis
IL	Interleukin
MVD	Mevalonate diphosphate decarboxylase
MVK	Mevalonate kinase
PMKV	Phosphomevalonate kinase
PSEK	Progressive Symmetrical Erythrokeratoderma
RCTs	Randomized controlled trials
SC	Stratum corneum
STS	Steroid sulfatase
TEWL	Transepidermal water loss
UV	Ultraviolet
XLRI	Recessive X-Linked ichthyosis
